# Epistemological Tensions in the EU HTA Joint Clinical Assessment: The Illusion of Judgment-Free Evaluation

**DOI:** 10.3390/jmahp14030040

**Published:** 2026-07-24

**Authors:** Mondher Toumi, Bruno Falissard, Asma Jouini, Imen Soussi, Steven Simoens, Maarten Postma, Laurent Boyer, Borislav Borissov, Renato Bernardini, Stefano Capri, David Danko, Claude Dussart, Frank-Ulrich Fricke, Pascal Auquier

**Affiliations:** 1 CEReSS/UR3279—Health Services Research and Quality of Life Center, Aix-Marseille University, 13385 Marseille, France; laurent.boyer@univ-amu.fr (L.B.); pascal.auquier@univ-amu.fr (P.A.); 2 CESP, INSERM U1018, Université Paris-Saclay, 94800 Villejuif, France; bruno.falissard@gmail.com; 3 Clever Access, Tunis 1058, Tunisia; asma.jouini@clever-access.com (A.J.); imen.soussi@clever-access.com (I.S.); 4 Department of Pharmaceutical and Pharmacological Sciences, Katholieke Universiteit Leuven, 3000 Leuven, Belgium; steven.simoens@kuleuven.be; 5 Department of Health Sciences, University Medical Center Groningen, University of Groningen, 9700 AB Groningen, The Netherlands; m.j.postma@rug.nl; 6 Department of Economics, Econometrics & Finance, Faculty of Economics & Business, University of Groningen, 9713 AB Groningen, The Netherlands; 7 Center of Excellence in Higher Education for Pharmaceutical Care Innovation, Universitas Padjadjaran, Bandung 40132, Indonesia; 8 Department of Health Technology Assessment, Faculty of Public Health, Medical University of Sofia, 1527 Sofia, Bulgaria; borislav.borissov@prescriptia.com; 9 Section of Pharmacology, Department of Biomedical and Biotechnological Sciences (BIOMETEC), University of Catania, 95124 Catania, Italy; bernardi@unict.it; 10 School of Economics and Management, Cattaneo-LIUC University, 21053 Castellanza, Italy; scapri@liuc.it; 11 Ideas & Solutions Ltd., H-1114 Budapest, Hungary; david.danko@i-s.hu; 12 UR 4129 P2S Parcours Santé Systémique, Université Claude Bernard Lyon 1, 7-11 Rue Guilllaume Paradin, 69008 Lyon, France; claude.dussart@chu-lyon.fr; 13 Pharmacie Centrale, Hospices Civils de Lyon, 57 Rue Francisque Darcieux, Saint-Genis-Laval, 69230 Lyon, France; 14 Faculty of Business Administration, Technische Hochschule Nürnberg, 90489 Nürnberg, Germany; frank-ulrich.fricke@th-nuernberg.de

## 1. Introduction

The practice of Health Technology Assessment (HTA) inherently involves moral, epistemological, and ontological commitments, which shape how assessments are conducted, interpreted, and used for decision-making [1,2].

Moral commitments concern what makes a health technology desirable or acceptable—for example, considerations of beneficence (doing good), nonmaleficence (avoiding harm), autonomy (respecting patient choices), and justice (fair distribution of resources) [1,2,3]. Complementing this moral framework, the European Commission Expert Panel on Effective Ways of Investing in Health has proposed four core principles of value-based care that capture the broader social and moral impact of health technologies: personal value (alignment with patients’ individual goals and preferences), technical value (optimal use of allocated resources, avoiding waste), allocative value (equitable distribution of resources across patient populations), and societal value (contribution to social participation and cohesion) [4].

Epistemological commitments in HTA relate to how reliable knowledge about health technologies is obtained and validated [5,6]. HTA practitioners commit to specific norms about evidence quality, study design, and methods of data analysis and synthesis, which influence what counts as valid or trustworthy information. This includes assumptions about the type of evidence prioritized (e.g., randomized controlled trials), how uncertainties are handled, and how evidence translates from clinical trials to real-world settings [5,6]. A complementary philosophical perspective relevant to the epistemological foundations of HTA is Karl Popper’s critical rationalism, which holds that scientific claims can never be definitively proven true but only rigorously tested and potentially falsified [7,8]. Popper argued that scientific knowledge advances through a process of conjectures (proposed hypotheses or theoretical explanations) and refutations (systematic attempts to disprove those hypotheses), whereby hypotheses are subjected to severe attempts at falsification. In doing so, Popper challenged the logical justification of induction (the reasoning process by which general conclusions are derived from repeated observations), which underpins empirical research and statistical inference, including both frequentist and Bayesian approaches. While empirical evidence may corroborate (provide supporting evidence for, without definitively proving) a theory by withstanding such tests, it can never conclusively verify it or establish it as permanently true [7,8]. This perspective reflects a fallibilist view (the philosophical position that any knowledge claim may ultimately be mistaken) of scientific knowledge, recognizing that all empirical claims remain provisional and contingent (dependent on underlying assumptions and methodological choices). Within the context of HTA, where conclusions regarding relative effectiveness and value are derived from clinical trials, real-world evidence, and statistical modeling, this implies that such conclusions cannot be considered epistemically final but remain subject to revision and reinterpretation as new evidence or alternative methodological approaches emerge. The reliance on evidentiary thresholds, outcome selection, and uncertainty management therefore reflects not only technical procedures but also epistemological and normative considerations. By emphasizing the revisable and value-informed nature of empirical inference, critical rationalism challenges strong technocratic assumptions (the belief that technical methods alone can produce fully objective and value-free decisions) and underscores that HTA cannot be a purely mechanistic (entirely automatic and rule-based) or judgment-free process, but must remain transparent about its assumptions and open to ongoing methodological refinement.

Ontological commitments concern what effects and outcomes of health technologies are considered real, relevant, or conceivable in the assessment [1]. This involves decisions about which health states, patient experiences, or societal impacts are included or excluded from evaluation. For example, HTA must decide whether to consider only direct clinical outcomes or also broader social, psychological, or environmental effects. These commitments define the “reality” that HTA engages with and delimit the phenomena that can be meaningfully assessed and acted upon [9,10].

In summary, these are moral commitments about what is good, just, and acceptable in health care technologies; epistemological commitments about how to generate and interpret reliable evidence; and ontological commitments about what effects and outcomes are real and relevant to assess. These commitments shape the normative foundation of HTA and highlight the importance of explicitly addressing values alongside empirical evidence in health technology decision-making [1,3].

These three types of commitments are intertwined and normative, meaning HTA is not a purely objective or technical exercise but one that involves value-laden decisions at every stage [1,2,3,11]. Recognizing these commitments supports the integration of normative analysis and stakeholder participation, fostering transparency and shared understanding among assessors, decision-makers, and affected populations [1,2,3]. This approach helps ensure that HTA outcomes are both scientifically credible and ethically justified, promoting equitable and socially relevant health policies. This is why considering these three commitments explicitly when developing a new HTA decision analysis framework model is unavoidable. Analyzing these commitments is important to understanding the underlying foundation of the HTA analysis framework and its consistency in the light of the objectives it pursues.

The recent European Union (EU) regulation on HTA introduces the Joint Clinical Assessment (JCA) as a harmonized collaborative approach to assessing the relative effectiveness of health technologies across Member States (MS). A core body was established to implement the EU HTA Regulation, the MS Coordination Group on Health Technology Assessment (HTACG). While the JCA is designed to be factual and free of value judgments, its epistemological foundations raise significant concerns [12].

No foundational epistemological, moral, or ontological commitments were made publicly available, which is an important issue from the transparency perspective and for the consistency of the proposed JCA model. These commitments are implicit in the EU-HTA Regulation, its implementing act, and documents from the HTACG. This paper critically examines the JCA process from an epistemological perspective, highlighting the decision-making, ontological implications, and impact on the selected design. It argues that the methodological framework underpinning the JCA, which stems from its historical design, does not recognize epistemological principles, thereby compromising the transparency and consistency of its outputs.

For clarity, the various epistemological topics are presented separately, although they are interconnected and frequently influence one another.

## 2. The PICO Framework (Population, Intervention, Comparator, and Outcome)

The PICO framework is central to JCA scoping and outcomes [13]. It fundamentally shapes the epistemological framework of EU HTA, showing HTACG’s preference for a positivist, experimental approach over a constructivist, ethical one, as seen in the PICO generation exercise performed by the JCA subgroup [14]. This is an impactful epistemological choice.

PICO rigidly prespecifies research questions, limiting evidence synthesis to predefined parameters. This excludes outcomes, populations, comparators, or ways of using the intervention not articulated in the initial scope and potentially omits real-world clinical nuances. The PICO framework within the EU HTA process prioritizes quantifiable outcomes (e.g., mortality rates, morbidity rates, etc.) over qualitative patient experiences [15].

Through its link to the Hierarchy of Evidence, the framework implicitly elevates comparative effectiveness data as the primary epistemic authority [5]. It mandates direct comparisons with existing alternatives, thereby reinforcing a rigid Hierarchy of Evidence. While indirect treatment comparisons are possible, they are placed low in the hierarchy. Furthermore, PICO introduces study design ranking by favoring randomized controlled trials (RCTs), which may not reflect real-world potential contribution.

Translation challenges arise as PICO clashes with clinical trial frameworks like estimand [16]. While estimand is clinical trial-focused and aims to inform internal, external validity, and causal relationship, PICO is HTA-focused and aims to inform relative effectiveness. RCTs are mainly constructed for regulatory purposes and may misalign with the PICO framework [16,17,18]. While the guidance on statistical analysis recommends sensitivity analysis [19], it is unclear how intercurrent events may be integrated within the PICO framework unless a new PICO is developed.

There is also a risk of reductionism. Fragmenting assessments into discrete PICO components may obscure complex interactions, such as comorbidities and social factors, hinder the understanding of unanticipated findings, and oversimplify multidimensional health science into a series of binary comparisons.

Moreover, the PICO framework limits the ability of patients to contribute their experiences and preferences during the assessment phase [20,21]. It offers a rigid structure that conflicts with the EU HTA Regulation, which mandates broader methodological approaches beyond RCTs, especially for novel therapies like advanced therapy medicinal products (ATMPs) and vaccines.

Despite these epistemological limitations, PICO provides a highly standardized framework, which continues to be a key feature in the JCA process. Most HTA agencies use the PICO framework explicitly or implicitly. While the United Kingdom National Institute for Health and Care Excellence (NICE) has a formal scoping process, French, German, Italian, and Spanish HTA agencies do not. In Germany, early consultation with the Federal Joint Committee (G-BA) allows the definition of the population, relevant comparator(s), and outcomes and subgroups; however, it is not a mandatory step. As a result, in these countries, any relevant evidence meeting general HTA requirements can be submitted, regardless of predefined PICOs. Predefined PICOs limit evidence submission flexibility.

## 3. Multiplicity of PICOs and Epistemic Uncertainty

The JCA scoping process consolidates individual MS PICOs, often resulting in an excessive number [13]. Recent exercises by the JCA subgroup identified at least 13 PICOs for each of two oncology products, with the comparator being the most frequent source of differences between PICOs [14]. It is unlikely to address this number of PICOs through direct randomized evidence. Typically, only one phase III clinical trial with at best one active comparator is available at launch time [22]. As a result, indirect treatment comparisons (ITCs) are unavoidable. The HTACG ranked non-randomized evidence, particularly ITCs, low in the evidence hierarchy [5,23]. Consequently, many PICOs will have the lowest certainty of relative effectiveness due to the epistemological standards set by the HTACG.

## 4. Multiplicity of Hypotheses and Type I Errors

The hypothetico-deductive model adopted by the HTACG is also known as the Neyman–Pearson testing framework, named after the authors who described and proposed this model [19], which is designed to control Type I errors (false positives) while maximizing test power for a single-hypothesis test [24]. However, when multiple hypotheses or endpoints are tested simultaneously, this framework faces the problem of multiplicity due to multiple comparisons [25]. The JCA guidance requires the testing of all endpoints (e.g., adverse events at the organ level regardless of frequency, seriousness, or severity), leading to hundreds of hypothesis tests per PICO [19]. This inflates Type I errors [26]. While it mandates reporting the 1-alpha Confidence Interval and estimates for each endpoint, it does not address the challenge of accounting for multiplicity, presenting an epistemological difficulty for the interpretation of the finding. There is therefore an epistemological contradiction between adopting the hypothetico-deductive model—the Neyman–Pearson testing framework, and the requirement for testing multiple outcomes without addressing the alpha inflation and multiplicity issue clearly in the guidance.

## 5. Post Hoc Analysis and Nominal *p*-Values

As PICOs will be defined after the evidence is analyzed and submitted to the JCA [13], all PICOs not predefined in the statistical analysis plan will be considered part of post hoc analysis [19]. This will apply to the vast majority of PICOs. The hypothetico-deductive model prohibits calculating or reporting *p*-values for post hoc analysis [27]. However, under the assumption that post hoc analysis may be more important for some MS than ad hoc analysis, the HTACG proposes calculating the *p*-value and labeling it “nominal”, without reference to a commonly accepted definition [19], further undermining the epistemic rigor of the process.

In statistics, the nominal *p*-value has emerged as a term to describe a *p*-value uncorrected for multiple comparisons, although it is generally considered inappropriate or misleading in standard statistical textbooks. Indeed, according to statistical principles, unadjusted *p*-values for multiple testing should not be reported, and *p*-values for post hoc analyses violate the hypothetico-deductive model, which holds that hypothesis testing should be defined a priori and not computed retrospectively (i.e., in post hoc analyses) [28,29]. For instance, the French National Health Authority (HAS) is very inflexible about the requirement to adjust for multiplicity analysis in its doctrine [30].

## 6. Ontic Component and Judgment-Free Assessment

The JCA equates judgment-free with decontextualized assessment, aiming to evaluate the ontic component of clinical evidence. The ontic component refers to the objective, mind-independent aspects of the experimental system, such as real features, structures, and causal mechanisms that exist independently of observers or interpretations [31,32,33]. Assessing the ontic component requires a reference model or structure against which evidence is compared. Models and structures simplify reality and evolve with knowledge development [34,35]. The outcome of such an assessment is binary: the evidence either matches or does not match the model or structure [36]. However, assessing ontic components without any judgment is impossible because ontic assessment inherently involves interpretive and normative elements [31,37,38,39].

The JCA relies on a combination of methodological guidelines (rules on how to conduct assessments) and guidance documents (non-binding expert advice) as its reference framework. This hybrid fails to provide a coherent ontic structure, as guidance documents lack enforceability and often rely on arbitrary consensus rather than empirical validation or systematic literature reviews. Additionally, these guidance documents and guidelines have been challenged elsewhere already [40,41,42,43].

Moreover, ontic assessment cannot be judgment-free. Interpreting evidence within any model necessarily involves normative choices. Thus, JCA’s ambition to produce a purely factual, decontextualized output without normative evaluation is both epistemologically and ontologically not an option.

The closest HTA process to JCA is that outlined by the German independent Institute for Quality and Efficiency in Health Care (IQWiG). IQWiG guidelines are detailed, providing a clear reference for assessing deviations [44]. Although IQWiG does not perform appraisals, it recommends additional benefits to G-BA, which is prohibited within JCA [12,44].

## 7. Certainty, Uncertainty, and Epistemological Confusion

Scientific empirical reasoning operates predominantly under uncertainty, with certainty being a rare ideal. This asymmetry means uncertainty is the default epistemic condition, while certainty is exceptional [45]. When concluding that an intervention is superior to the comparator, we accept 5% probability of being wrong, so the outcome is not certain. In epistemology, certainty and uncertainty are not symmetrical concepts [46,47]; rather, they have an asymmetrical relationship grounded in how knowledge and doubt function. Yet the JCA treats certainty and uncertainty as symmetrical, converting probabilities into a quasi-continuum of certainty degrees. This conflation is evident in the European Commission publication on food safety assessment that advocates a 0–100% scale to communicate certainty, implying that 100% equates to absolute certainty—while the overall publication explains how to measure uncertainty but ultimately reports the level of certainty assuming symmetrical propriety of the two concepts. This notion is indefensible from an epistemological standpoint in empirical sciences [48].

The JCA aims to report the “degree of certainty” in relative effectiveness. This leads to overconfidence in conclusions derived from uncertainty assessments and incorrectly assumes symmetry with certainty.

## 8. Exclusionary and Authoritative Nature of the EU HTA Process

The JCA process evaluates if evidence submitted by the Health Technology Developer (HTD) aligns with guidelines and guidance documents [12]. In fact, JCA assessors will measure any deviation from the methodological guidance and guidelines. As a factual exercise, it remains, according to the guidance documents, decontextualized and judgment-free, leaving little room for input from patients, clinicians, or experts experiences. The HTD cannot participate in any part of the process [13,49]. They may only report factual errors and are not allowed to challenge JCA scoping or reports. As long as the guidance and guidelines remain unchanged, there is little to no opportunity for third parties to meaningfully influence the outcome.

The only opportunity for patients or clinical experts to impact JCA is to influence the PICO. They are not part of the discussion of PICOs at the different stages of development [13], but they are presented with the final conclusions and allowed to comment with little chance to impact the final PICOs.

A stakeholder network has been established to provide input and recommendations to the process and guidelines/guidance documents. However, early feedback indicates the process is slow-moving and that even consensual recommendations are difficult to implement.

Focusing heavily on internal validity and RCT can result in the exclusion of certain patient subgroups from clinical trials, which may unintentionally bias assessments against marginalized or vulnerable populations, potentially coming into conflict with HTA moral commitments [10,11,50,51].

The guidance demonstrates a strong commitment to empirical rigor, yet it overlooks essential qualitative evidence such as patient preferences, exit interviews from clinical trials, patient experiences, and real-world evidence, which are crucial for specific situations [52,53]. Prioritizing quantifiable metrics overlooks qualitative outcomes such as dignity and caregiver burden, indicating an ontological preference for measurable phenomena.

These above-described JCA features make the JCA process non-inclusive and authoritative in several ways by design.

## 9. Consolidation of Assessment

While there are recommendations for individual outcomes assessment, it is unclear how certainty of relative effectiveness at the outcome level will be consolidated at the PICO level. Only basic information is provided for consolidating the risk of biases at the PICO level. Ranking outcomes is prohibited by EU HTA law [12]. Thus, JCA must treat a non-severe, non-serious extremely rare adverse event with the same importance as mortality.

The absence of a normative ontological commitment, that is, a clear stance on the desirability or relevance of outcomes, undermines one of the three epistemological pillars of HTA. It also makes the assessment process burdensome, as all outcomes and endpoints must receive equal attention. Notably, adverse events will be reported in large numbers in the JCA submission and will be subject to statistical testing. Given the volume of data, limited resources, and time constraints, some reported events are likely to go unassessed. This inevitably leads to the omission of certain endpoints, despite their formal inclusion in the submission.

## 10. The Degree of Certainty

The degree of certainty of relative effectiveness is generally not high due to the inherent imperfections in life sciences experiments [45,54]. Researchers strive to balance complexity, feasibility, cost, time, and reliability of outcomes, often making compromises to attain empirically valid results. While HTD will focus on key endpoints, JCA will treat all endpoints equally irrespective of their importance. This approach will disregard the HTD rationale aimed at minimizing biases on key endpoints, while potentially accepting higher uncertainty regarding less critical ones as a trade-off. In practice, a perfect experiment is unattainable [55,56]. Given the multiplicity of outcomes and assessments at the outcome level, combined with impractical and high-bar methodological requirements:

- High certainty of relative effectiveness will be exceptional.
- Medium certainty may sometimes be attainable.
- Most PICOs will have a low certainty rating.

This presumes that the guidance and scope of the JCA will be adhered to and not subject to revision. This suggests a floor effect for the degree of certainty of relative effectiveness assessment, leaving the HTACG focusing on the tables of results with limited interest in the degree of certainty.

The decision to include ATMPs and approximately 50% of orphan-designated products, many in oncology or emerging therapeutic classes, further contributes to low certainty. These are areas where clinical knowledge is still evolving, or the rarity of the condition makes usual large-size RCT less feasible, inherently limiting the degree of certainty achievable in assessments.

If the vast majority of PICOs are ranked as having a low degree of certainty of relative effectiveness, the usefulness of this rating will be limited.

## 11. Accelerated Versus Standard Procedure

The HTACG distinguishes between two timelines: the accelerated procedure and the standard procedure. The accelerated process applies to variations such as extensions of indication and aligns with the European Medicines Agency (EMA) timelines [12,49]. From a regulatory standpoint, shortening timelines in these cases is logical because certain aspects—such as quality, toxicology, pharmacology, and phase I studies—may not require a new review, thereby reducing the assessment workload significantly.

However, from an HTA perspective, the clinical evaluation for an indication extension is just as rigorous and demanding as that of the initial application. This raises an epistemological question about the rationale behind having two different timelines for essentially the same process and workload. Since the standard timeline is already considered tight, it is important to clarify whether the accelerated process is truly realistic and feasible in practice [57].

From an epistemological standpoint, having two separate timelines is unjustified. This dual timeline arrangement arises from an arbitrary attempt to align regulatory and JCA deadlines, without adequately considering feasibility.

## 12. The Hierarchy of Evidence

The Hierarchy of Evidence, though not explicitly addressed, is clear in guidance documents [5,23]. Randomized clinical trials are at the top, with other types of evidence at the bottom [5,23]. Non-randomized evidence, despite some nuances like anchored versus unanchored indirect treatment comparisons, generally occupy the lower end of the scale [5,23]. The hierarchy appears dichotomic rather than appearing as a continuum in JCA guidance documents.

Guidance documents recommend prioritizing robust evidence, ignoring less robust sources when both co-exist for the same PICO, and favor direct evidence over combining direct and indirect evidence [5,23,58]. This is inconsistent with evidence-based medicine (EBM) philosophy, which defines different levels of evidence, ranks them along a continuum, and considers all evidence [59].

RCTs are positioned at the top of the evidence hierarchy, which aligns with evidence-based medicine (EBM) and the majority of HTA guidelines. However, the epistemic basis for this placement and its broader implications have not been addressed.

## 13. Epistemological Perspective of Guidance Documents

The guidance documents have been heavily criticized [40,41,42,43,60]. Its publication led to European Access Academy (EAA) faculty members addressing an open later to the European Commission, which raised the issues of the low applicability of the HTACG and the imposition of principles of EBM on MS by the HTACG and concluded as follows: “Considering the current status of adopted methodological guidance documents an additional major aspect of uncertainty needs to be addressed: the uncertainty of validity of recommended assessment methods. The EAA Faculty would like to express our profound concern that the evidence base for the currently adopted EU HTA methodological framework does not reach beyond an informal consensus among the involved members of the CG. Major uncertainty regarding the applicability, robustness and validity of those methods prevails” [61].

Critics, including the European Access Academy, have flagged serious epistemological flaws in JCA guidance documents:

- Erroneous definitions of internal and external validity [5,43].
- Oversimplified external validity assessment [5,62].
- Inadequate handling of multiplicity [19,40].
- Acceptance of nominal *p*-values in post hoc contexts [19,40].
- Poor management of missing data [19,40].
- Minimum Clinically Important Difference (MCID) is not addressed within the JCA [5] while it should be reported. It may be reasonable to ask HTDs to document MCID for critical endpoints.
- Continued use of the outdated Risk of Bias (RoB) 1 tool rather than the updated RoB 2 [63].

Additional arbitrary practices include:

- Excluding Embase (a medical literature database) and conference abstracts from systematic literature reviews (SLRs) [63].
- Requiring a shifted null hypothesis (altering the baseline assumption) in population-adjusted indirect treatment comparisons (PAICs) [41].
- Relying solely on narrative assessments for external validity [43].

Therefore, using scientifically inadequate methodological guidance documents and guidelines as the reference to assess how much submitted evidence deviates from this reference is invalid from an epistemological perspective.

Although the guidance documents avoid defining norms regularly, two quantitative norms are defined: a high correlation for surrogate endpoint validation at 0.85 and a “large effect size” norm, five times the effect of the reference health technology intervention. Both are unrealistic in medical sciences.

This makes the guidance documents challenging to implement and raises doubts about the JCA report’s reliability.

## 14. The Source of HTA Epistemological Contradictions

The epistemological contradiction is seen in HTA across different countries. HTA deals with regulatory requirements output for marketing authorization and considerations for reimbursement and pricing, each following different epistemological frameworks. Marketing authorization involves broad evidence beyond clinical trials such as biological and medical sciences disciplines including animal models, in vitro models, pharmacokinetics/dynamics, and mechanisms of action, for example. Reimbursement and pricing fall under the category of social sciences [64]. HTA intersects these two fields and faces challenges in determining its alignment [65,66]. Many HTA assessors have a background in experimental life sciences and may not consider social sciences, which are important for providing essential information in decision sciences.

Epistemological awareness is crucial for methodological choices and understanding HTA’s policy role. HTA should support reimbursement and pricing decisions by considering funding community expectations and patient preferences, rather than repeating the marketing authorization clinical assessment process. The aim is to satisfy population needs with the most appropriate epistemological model.

## 15. Conclusions

Epistemological ontological decisions guide the assessment of evidence in the HTA process [1,66]. It is important to acknowledge that, while peer-review evaluation is generally more thorough than a single evaluation, assessors may also have cognitive biases and conflicts of interest, which might not always be fully disclosed, either intentionally or unconsciously. This may further confuse the process.

To ensure a transparent evaluation, HTD, patients, and clinical experts should play a larger role in the JCA, allowing the consideration of multiple perspectives. The results of such evaluations should offer a balanced explanation of differing viewpoints and judgments, enabling decision-makers (i.e., MS) to make informed decisions based on their own criteria, which theoretically reflect a political and democratic process.

To ensure alignment between 27 MS and ensure a consistent epistemological HTA framework, there is a need to a priori align on the HTA’s purpose and the three commitments (moral, epistemological and ontological) that will drive the development of guidance and guidelines. This will ensure a higher level of consistency and awareness of the ultimate methodological choice implications driving the JCA. JCAs’ guidance documents and guidelines have been developed while looking at the minimum consensus, ignoring the underlying epistemological, moral and ontological commitments.

JCA’s design contains multiple epistemological contradictions. Any assessment of relative effectiveness cannot be properly judged, as is clearly described in the context of the risk of bias assessment. The JCA attempts to evaluate the degree of certainty in terms of relative effectiveness through a process that achieves the following objectives:

- Multiplies PICOs beyond reasonable empirical feasibility;
- Fails to adequately address Type I errors due to multiplicity;
- Treats post hoc analyses as hypothesis-driven evidence;
- Relies on non-enforceable and epistemically weak, imprecise and inconsistent guidance documents;
- Excludes normative, moral, and contextual judgments;
- Operates under a misconceived notion of certainty.

These foundational issues make the JCA process unfit for its intended purpose. Rather than promoting harmonization and robust decision-making, it introduces new layers of epistemological, ontological, and ethical confusion. Future development steps of the EU HTA methodological framework should aim to primarily align with the foundational epistemological, ontological and ethical principles of science, medicine, and public health policy. This requires a comprehensive understanding of epistemology and clear alignment on the societal purposes of HTA. This process will help in reaching a robust and stable methodological consensual framework.

## Data Availability

No new data were created or analyzed in this study. Data sharing is not applicable to this article.

## Abbreviations

The following abbreviations are used in this manuscript:

ATMP	Advanced Therapy Medicinal Products
EAA	European Access Academy
EBM	Evidence Based Medicine
EU	European Union
G-BA	Gemeinsamer Bundesausschuss (Federal Joint Committee, Germany)
HAS	French National Health Authority
HTA	Health Technology Assessment
HTACG	Health Technology Assessment Coordination Group (of the EU)
HTD	Health Technology Developer
IQWiG	Institut für Qualität und Wirtschaftlichkeit im Gesundheitswesen (Institute for Quality and Efficiency in Health Care, Germany)
ITC	Indirect Treatment Comparison
JCA	Joint Clinical Assessment
MCID	Minimum Clinical Important Difference
MS	Member States (of the EU)
NICE	National Institute for Health and Care Excellence (United Kingdom)
PAIC	Population-Adjusted Indirect Treatment Comparisons
PICO	Population, Intervention, Comparator, Outcome
RCT	Randomized Controlled Trial
RoB	Risk of Bias
SLR	Systematic Literature Review
